# Clinical Mass Spectrometry – Call for Papers

**DOI:** 10.1016/j.clinms.2016.08.002

**Published:** 2016-08-20

**Authors:** Alan L. Rockwood, Michael Vogeser

We are pleased to announce the international journal, *Clinical Mass Spectrometry* (CMS), to the scientific and clinical community. **CMS** is the first journal specifically devoted to mass spectrometric technologies in the context of their current and future application in clinical diagnostics. CMS is the official journal of the not-for-profit *Association for Mass Spectrometry: Applications to the Clinical Laboratory* (**MSACL**, www.msacl.org), founded in 2009.

Mass spectrometers are fundamental analytical tools that offer not only classical target analyte identification and quantitation, but also extend to applications as diverse as tissue imaging, rapid bacterial identification, and real-time tissue analysis. During the past decade, mass spectrometric technologies have contributed key elements of innovation to clinical pathology, evidenced by the increasing number of publications focused on mass spectrometry in journals devoted to clinical laboratory science.

Unlike other journals, CMS will present focused and dedicated coverage, addressing the state-of-the-art in this rapidly growing field while supporting both general and sophisticated competencies. A powerful adjuvant to the continued development and implementation of the technology will be the comprehensive and interdisciplinary composition of the CMS Advisory and Editorial Boards, which include scientists from diverse backgrounds who are all excited to support the growth of the field. By leveraging networks established through MSACL, the journal will accentuate and support educational initiatives and intellectual dissemination, with the foremost interest being patient welfare.

CMS will publish original research articles, reviews, letters to the editors, troubleshooting tips, best practices documents, and special issues, as well as concise technical reports essential for the development of our field. Additionally, the journal will facilitate interaction with regulatory bodies, as well as between manufacturers and users.

CMS will be an electronic-only subscription and open access (hybrid) journal; although to establish initial awareness and traction, published articles will be available without restriction through 2016. CMS is published by Elsevier, hence, authors will realize extensive exposure through the online platform, ScienceDirect.

We invite you to submit your work to this ground breaking journal, and eagerly encourage feedback as we move forward with the goal of working with the community to foster the measured development and application of this elegant technology to shift the face of clinical analysis and, ultimately, the practice of medicine for the benefit of humanity.

